# A longitudinal evaluation of *Treponema pallidum* PCR testing in early syphilis

**DOI:** 10.1186/1471-2334-12-353

**Published:** 2012-12-17

**Authors:** Matt Shields, Rebecca J Guy, Neisha J Jeoffreys, Robert J Finlayson, Basil Donovan

**Affiliations:** 1 Taylor Square Private Clinic, Darlinghurst, NSW, 2010, Australia; 2 Kirby Institute, University of New South Wales, Sydney, NSW, 2052, Australia; 3 Centre for Infectious Diseases and Microbiology Laboratory Services (CIDMLS), Institute of Clinical Pathology and Medical Research (ICPMR), Westmead Hospital, Westmead, NSW, 2145, Australia; 4 Sydney Sexual Health Centre, Sydney Hospital, Sydney, NSW, 2000, Australia

**Keywords:** Syphilis, PCR, Evaluation

## Abstract

**Background:**

Syphilis is a growing public health problem among men who have sex with men (MSM) globally. Rapid and accurate detection of syphilis is vital to ensure patients and their contacts receive timely treatment and reduce ongoing transmission.

**Methods:**

We evaluated a PCR assay for the diagnosis of *Treponema pallidum* using swabs of suspected early syphilis lesions in longitudinally assessed MSM.

**Results:**

We tested 260 MSM for *T pallidum* by PCR on 288 occasions: 77 (26.7%) had early syphilis that was serologically confirmed at baseline or within six weeks, and 211 (73.3%) remained seronegative for syphilis. Of 55 men with primary syphilis, 49 were PCR positive, giving a sensitivity of 89.1% (95% CI: 77.8%-95.9%) and a specificity of 99.1% (95% CI: 96.5%-99.9%). Of 22 men with secondary syphilis, 11 were PCR positive, giving a sensitivity of 50% (95% CI: 28.2%-71.8%) and a specificity of 100% (95% CI: 66.4%-71.8%). Of the 77 syphilis cases, 43 (56%) were HIV positive and the sensitivity and specificity of the PCR test did not vary by HIV status. The PCR test was able to detect up to five (10%) primary infections that were initially seronegative, including one HIV positive man with delayed seroconversion to syphilis (72 to 140 days) and one HIV positive man who did not seroconvert to syphilis over 14 months follow-up. Both men had been treated for syphilis within a week of the PCR test.

**Conclusions:**

*T pallidum* PCR is a potentially powerful tool for the early diagnosis of primary syphilis, particularly where a serological response has yet to develop.

## Background

Syphilis is a sexually transmissible infection (STI) caused by *Treponema pallidum*. The infection is systemic, usually involving mucocutaneous ulcers and rashes in the early phases, and a range of serious complications including cardiovascular and neurological disease in later phases [1,2].

As in many countries, Australian men who have sex with men (MSM) with HIV infection are disproportionately affected by syphilis. Although only ~10% of Australian MSM have HIV infection they contribute around half of all cases of infectious syphilis among MSM [3,4]. Community-based cohort studies have shown that the incidence of syphilis in MSM with HIV infection is 5–10 times higher than in MSM without HIV [5]. Reasons for this differential are probably behavioural, [6]. but a biological phenomenon linked to HIV’s effect on the immune system has yet to be excluded [7].

Syphilis is at its most infectious in its primary and secondary stages, so early detection has been modelled to have the maximum benefit for preventing its onward transmission [8]. The interpretation of syphilis serology can be problematic in primary syphilis because serological responses might be delayed, often requiring the patient to return for re-testing.

Dark-field microscopy of secretions from chancres can provide a rapid point-of care test result, but the sensitivity is limited and the skill and meticulous care required for specimen collection and microscopy are rarely available. The loss of expertise in dark-field microscopy was lamented over 60 years ago [9].

HIV infection is more likely to be associated with exaggerated and delayed serological responses to syphilis infection [10]. This problem can result in a syphilis diagnosis being missed or the treatment being delayed, with ongoing transmission. A PCR based swab test has the potential to provide earlier diagnosis of primary syphilis in MSM with HIV and also demonstrate re-infection in high titre rapid plasma reagin (RPR) serofast MSM with HIV who have been previously treated for syphilis. Considering the high incidence of early syphilis in HIV-infected MSM, a more sensitive and robust direct test for T pallidum such as PCR is needed.

In this study we evaluated the performance of a probe based PCR assay used to detect *T pallidum* in suspected lesions of early syphilis, based on a comprehensive clinical and serological assessment.

## Methods

### Study setting and population

Taylor Square Private Clinic was established in 1981 as Australia’s first private sexual health clinic and has one of the highest case-loads of HIV-infected MSM patients in Australia [11]. All high-risk patients are routinely screened for *T pallidum*, *Chlamydia trachomatis*, *Neisseria gonorrhoeae* and HIV according to guidelines [12]. All patients in this study were consecutive high-risk MSM with lesions suggestive of early syphilis between 2004 and 2010, though in the early years of the study the test was in limited supply so it was only used for the highest-risk men. Heterosexual patients were excluded because syphilis is rare in heterosexuals in Australian cities. There was no standardized specimen collection protocol and several different doctors collected specimens.

### *T pallidum* PCR

#### Specimen collection

A dry swab specimen was obtained from genital, anal or oral ulcers; or from the surface of rashes, papular lesions, or mucosal erosions suggestive of secondary syphilis; then placed in transport medium and sent at room temperature to the Institute of Clinical Pathology and Medical Research (ICPMR) for syphilis PCR testing. Blood for syphilis serology was collected at the same time.

#### DNA extraction

DNA was extracted from clinical specimens using the NucliSENS easyMAG (BioMeurieux) total nucleic acid extraction system with off board lysis. In brief, swabs were placed in individual lysis tubes, vortexed vigorously and incubated at room temperature for 10 minutes. Lysis solution was then combined with magnetic silica in an extraction cartridge and DNA extracted in the easyMAG using Boom technology. DNA extracts were stored at −80°C prior to PCR testing.

#### Assay design

The *T pallidum* PCR targets a 146 base pair region of the gene encoding the 47kDa lipoprotein (Genbank accession number: M88769). The assay uses a 6 carboxyfluorescein (FAM) labelled hydrolysis probe and end point fluorescence detection to identify amplification of products.

The primers [Syph1: _1130_AGG GGA AGG TGC TGA CCA TAG_1150_; & Syph2: _1275_GGG AGT GAA ATC CGC AGA GAG_1255_] and hydrolysis probe [_1166_(6-FAM) AGC CTA AGC TTG TCA GCG ATC AAG C (BHQ1)_1191_] were designed using Primer3 and manufactured by Sigma Aldrich.

The PCR was performed in a 50uL reaction containing sample DNA, 3.5mM MgCl_2_, 200nM each primer, 100nM probe & HotStar Taq Master Mix (Qiagen) [containing 1.5mM MgCl_2;_ 200uM each dNTP & HotStar taq DNA polymerase]. Baseline fluorescence was determined using the FlourTracker (Stratagene) before amplification in a Eppendorf Mastercycler under the following conditions: 95°C × 15 min; 50 cycles of [96°C × 10 sec, 60°C x 1min, 72C × 1min]; 72°C × 5min. Post amplification fluorescence was again measured in the FluorTracker and the ratio of post- to pre-amplification fluorescence calculated.

Samples with fluorescence ratios greater than 3.4 were considered positive and confirmed by gel electrophoresis on a 2% agarose gel run for 40 minutes at 200V. Fluorescence ratios less than 1.5 indicated a negative sample, and samples with ratios between 1.5 and 3.4 were deemed equivocal and the assay repeated.

#### Controls

Individual samples were tested in parallel with inhibition and contamination controls and as both neat and diluted DNA to ensure detection of both weak and strong specimens. Inhibition controls consisted of sample DNA spiked with positive material, while contamination controls utilised no template reagent samples. Each run also contained a serial dilution of positive control to identify the limit of detection of the assay. The positive control was produced by extraction of DNA from a *T pallidum* (Nichols) suspension which had been previously used in the *T pallidum* immobilization assay. Initial experiments indicated the assay had a limit of detection of 10–100 organisms and was specific for *T pallidum* species.

### Syphilis serology

Sera were initially screened with fully automated one-step Immulite Treponemal EIA (Siemens Healthcare Diagnostics) which uses one major *T pallidum* recombinant antigen (p17). Specimens positive by EIA were then subjected to confirmatory testing with a RPR test (Reditest, Biokit) and a *T pallidum* particle agglutination assay (TPPA) (Serodia TPPA, Fujirebio) and fluorescent treponemal antibody absorption (FTA-ABS) test (MarDx**®**). Men that had previously been treated for syphilis were screened with the RPR test.

### Narrow definition of a syphilis case

A MSM patient was classified as having primary or secondary syphilis if:

1. His serology was positive by EIA and TPPA at the time of the PCR swab, after being negative in the previous year, or

2. His RPR titre had risen 4-fold (two titres) or more if he had previously been treated for syphilis.

However, as this definition excludes cases that are in the serological window period, for this study we used a broader case definition:

### Broader definition of a syphilis case

A MSM patient was classified as having primary or secondary syphilis if he had consistent symptoms and signs and:

1. His serology was positive by both the EIA and TPPA at the time of the PCR swab *or within 6 weeks of treatment*, after being negative in the previous year, or

2. His RPR titre rose 4-fold (two titres) or more if he had previously been treated for syphilis.

This expanded (longitudinal) definition of early syphilis was developed because many cases were treated presumptively (before serological confirmation) on clinical suspicion or as soon as a positive PCR test result became available. Direct detection tests such as dark-field microscopy [13] and PCR [14] for syphilis have been documented to sometimes precede the serological response, as with all infections.

We conservatively settled on a 6-week cut-off as the minimum period for someone to be (a) treated for syphilis, (b) to have a long-acting injected penicillin or a 2-week course of oral doxycycline ‘wash out’, (c) to become newly-infected with syphilis, and (d) to thereafter mount a serological response. Any seroconversion to syphilis within 6 weeks was, therefore, deemed to result from a *T pallidum* infection at the time of the initial swab collection and treatment.

### Uninfected case definition

A MSM patient was deemed to not have syphilis if the *T pallidum* PCR test was negative and:

1. His *T pallidum* EIA test remained negative, or

2. His RPR titre did not rise 4-fold (two titres) or more if he had previously been treated for syphilis.

### Analysis

Clinical and laboratory data that were extracted from the patient management system included HIV status, syphilis stage, treatment, previous syphilis, syphilis serology and PCR results.

The performance (sensitivity and specificity) of the *Treponema pallidum* PCR in lesions suggestive of primary syphilis and secondary syphilis was then assessed against the definitions of a syphilis case (above), and by HIV status. For the purpose of comparability with other studies, we also assessed the performance of the PCR test against the narrower definition of syphilis that was limited to base-line clinical signs and serology. We calculated 95% confidence intervals (CIs) using the binomial approximation method.

Using a Chi-square test we compared the performance of the PCR test in HIV-positive and HIV-negative men.

All analyses were conducted using Stata statistical software version 9 [15].

## Results

We collected swabs for *T pallidum* PCR testing from 260 MSM patients on 288 occasions over the 7-year study period. Of the 288 specimens, 35.8% were collected from penile ulcers, 36.1% from anal ulcers, 17.4% from oral ulcers, 9% from rashes, 1.4% from snail track ulcers of the mouth and 0.3% from perianal condylomata lata (Table 1).

**Table 1:** **Performance of*T pallidum*PCR by syphilis stage and lesion site**

**Category**	**Breakdown**	**PCR- pos/ serology- pos**	**Sensitivity (95% CI)**	**PCR- neg/ serology-neg**	**Specificity (95% CI)**
**Lesion stage**	Primary lesions^(a)^	49/55	89.1 (77.8-95.9)	200/202	99.1 (96.5-99.9)
	Secondary lesions^(b)^	11/22	50.0 (28.2-71.8)	9/9	100.0 (66.4-100)
**Lesion site**	Penile ulcer	30/33	90.9 (75.7-98.1)	68/70	97.1 (90.0-99.7)
	Anal ulcer	16/16	100.0 (79.4-100)	88/88	100.0 (95.9-100*)
	Oral ulcer	3/6	50.0 (11.8-88.2)	44/44	100.0 (91.9-100)
	Generalised rash	6/17	35.3 (14.2-61.7)	9/9	100.0 (66.4-100)
	Perianal condylomata lata/snail track ulcers of the mouth	5/5	100.0 (47.8-100)	-	-

(a)=penile, anal and oral ulcers.

(b)= Generalised rash, perianal condylomata lata, and snail track ulcers of the mouth.

### Performance of PCR against serology – broader definition

Using the broader case definition, 77 (26.7%) of the 288 PCR specimens were collected from men with primary or secondary syphilis at baseline and 211 (73.3%) were collected from men without syphilis.

Of the 55 men with primary syphilis, 49 were PCR positive, yielding a sensitivity of 89.1% (95% CI: 77.8%-95.9%) and a specificity of 99.1% (95% CI: 96.5%-99.9%). Of the 22 men with secondary syphilis, 11 were PCR positive, yielding a sensitivity of 50% (95% CI: 28.2%-71.8%) a specificity of 100% (95% CI: 66.4%-100%) (Table 1). By specimen type, the sensitivity was 90.9% (95% CI: 75.7%-98.1%) in penile chancres, 100% (95%CI: 79.4%-100%) in anal chancres, 50% (95%CI: 11.8%-88.2%) in oral chances, 35% (95%CI:14.2-61.7) in dry rashes of secondary syphilis, and 100% in moist secondary lesions (snail track oral lesions and perianal condylomata lata) (Table 1).

There was no significant difference in sensitivity and specificity of the PCR test, nor site or stage of syphilis, according to HIV status. Of the 77 syphilis cases, 43(56%) were HIV positive, including 53.7% of the primary cases and 62.5% of the secondary cases.

### Performance of PCR against serology – narrow case definition

Using the narrow case definition, three MSM who were TP PCR positive with primary syphilis who were initially seronegative but later seroconverted to syphilis (cases 1–3 in Table 2) were categorized as not having syphilis. This exercise netted a slightly higher sensitivity of 94.2% (95% CI: 84.1%-98.8%) than the broader case definition but a slightly lower specificity of 97.6% (95% CI: 94.4%-99.1%).

**Table 2:** Characteristics of patients that were PCR positive but seronegative for syphilis at baseline

**Case number**	**Clinical status**			**Baseline test results**			**Treatment**	**Intermittent negative EIA results**	**Follow-up positive serology tests**		**Syphilis seroconversion classification**
	**Site of ulcer**	**Prior syphilis**	**HIV status (CD4 cells/ul)**	**Syphilis PCR**	**HSV PCR**	**EIA/RPR/TPHA/FTA**	**Days since baseline**	**Days since baseline**	**Days since baseline**	**EIA/RPR/TPHA/FTA**	
1	Anus	No	N	P	N	N	14	-	7	P/N//P/P	Delayed
2	Penis and perianal	Yes	N	P	N	P/N/P/P	5	-	21	P/1:4/P/P	Delayed
3	Penis	No	N	P	N	N	11	7, 14	35	P/N/P/P	Delayed
4	Penis	No	P (774)	P	ND	N	6	14,42,72	170	P/N/P/P	Delayed or re-infection
5	Anus	No	P (646)	P	N	N	7	426	-	-	Persistent negative at 14 months

N=negative, P=positive, ND=not done, PCR=polymerase chain reaction, RPR= rapid plasma reagin, TPHA= treponema pallidum haemagglutination, FTA= fluorescent treponemal antibody, EIA=enzyme immunoassay, d=days, m=months.

### Discrepant results– PCR positive and serology negative

In five high-risk MSM the *T. pallidum* PCR test was positive and the syphilis serology was negative at baseline. The characteristics of these cases, including follow-up serology, are detailed in Table 2. Cases 1, 2, and 3 all seroconverted to syphilis within 35 days. Case 4 seroconverted between 72 and 170 days and was at ongoing risk, so it was not possible to determine whether he was a late seroconverter or was re-infected with syphilis. Case 5 remained seronegative after 14 months and received antibiotic treatment 7 days after sample collection. Both cases 4 and 5 were HIV positive with high CD4 counts. Notably, three men who seroconverted later, had not previously been treated for syphilis and remained seronegative in the RPR test.

## Discussion

We found the *T pallidum* PCR test to be both sensitive and specific in primary syphilis, thus facilitating rapid diagnosis and treatment. This evaluation has demonstrated that the sensitivity of *T. pallidum* was 89.1% for primary syphilis and 50.0% for secondary syphilis, including three infections in men that seroconverted to syphilis within five weeks. There was no difference in the sensitivity and specificity of the PCR test according to HIV status. The performance of *T pallidum* PCR testing in our evaluation was broadly comparable to previous studies using other PCR tests [14,16-19].

Previous evaluations have generally relied on baseline serology alone [16-18]. Clinicians are aware that the diagnosis of syphilis is not so straightforward and recognise that the diagnosis and staging of syphilis relies on a combination of knowledge of the individual’s past syphilis history and sexual behaviour, clinical appearance, and serological profile (including RPR titre and serological evolution), as well as direct detection techniques.

Like Heymans et al., [17] and Gayet-Ageron et al [16] we do not see much value in *T pallidum* PCR testing in suspected secondary syphilis. The sensitivity of PCR was modest (Table 3) and the sensitivity of both RPR and specific serological tests is virtually 100% in secondary syphilis [10]. That said, the sensitivity of the PCR test might be increased by improved specimen collection. There was a time when well-selected secondary lesions were lightly abraded with the edge of a large-bore needle or a scalpel to extract serum for dark-field microscopy [9] and this might also improve the sensitivity of PCR. This re-visited method of specimen-collection would be worth examining in future evaluations of *T pallidum* PCR tests. Notably, a study that assessed the performance of PCR using skin biopsy specimens to diagnose secondary syphilis found a higher sensitivity of 75% [19].

The lack of a standardised specimen collection protocol is a limitation of this study that may have reduced the apparent sensitivity of the PCR test. Another limitation of our study was that patient selection was not according to a formal protocol. Since the PCR test was in limited supply the test was used selectively and therefore we assume that the tested population was biased toward the higher risk MSM within the clinic. This bias might inflate the apparent specificity of the PCR test.

The PCR test seems likely to be most valuable in primary syphilis, where serology is more problematic than in secondary syphilis. Since HIV infection is more likely to be associated with exaggerated or delayed serological responses to syphilis, a PCR test has the potential to provide earlier diagnosis of primary syphilis in MSM with HIV and also demonstrate re-infection in high titre RPR serofast MSM with HIV who have been previously treated for syphilis. There were five (10% of the primary) cases in our evaluation where the PCR was positive and the serology was negative at baseline. In three cases the serology became positive between 7 and 35 days later. Palmer et al. also found two patients who were positive by PCR 12 and 21 days before seroconverting to syphilis, both were HIV positive [14]. A further two of our patients, who were HIV positive were not confirmed as becoming seropositive as a result of the current episode, though one was positive when next tested 7 months later. Palmer et al., also found a positive PCR result in a patient with HIV infection with consistently negative syphilis serology. This patient received a 10 day course of antibiotics one week after sampling. They hypothesized the prompt antibiotic treatment coupled with the patient’s immune dysfunction may have blunted a serological response to *T pallidum*[14].

## Conclusion

In conclusion, our evaluation demonstrated that *T pallidum* PCR could allow earlier diagnosis of primary syphilis, with potential clinical and public health benefits.

## Competing interests

The author(s) declare that they have no competing interests.

## Author's contributions

MS conceived the study, collected most of the data, conducted the first analysis, and produced the first draft of the manuscript. RJF was involved in data collection and drafting of the manuscript. NJ was responsible for the laboratory component and provided input to the manuscript. RJG provided epidemiological advice and assistance with drafting of the manuscript. BD provided clinical and research design advice, and assistance with drafting of the manuscript. All authors read and approved the final version of the manuscript.

## Pre-publication history

The pre-publication history for this paper can be accessed here:

http://www.biomedcentral.com/1471-2334/12/353/prepub
